# Putative adaptive inter-slope divergence of transposon frequency in fruit flies (*Drosophila melanogaster*) at “Evolution Canyon”, Mount Carmel, Israel

**DOI:** 10.1186/s13062-015-0074-5

**Published:** 2015-10-14

**Authors:** Avigdor Beiles, Shmuel Raz, Yuval Ben-Abu, Eviatar Nevo

**Affiliations:** Institute of Evolution, University of Haifa, Haifa, 31905 Israel; Department of Computational Biology, University of Haifa, Haifa, 31905 Israel; Projects and Physics Section, Sapir Academic College, D.N. Hof Ashkelon, 79165 Israel

**Keywords:** Transposons element, Climatic divergence, Adaptiveness, Evolution Canyon, Natural selection

## Abstract

**Background:**

The current analysis of transposon elements (TE) in *Drosophila melanogaster* at Evolution Canyon, (EC), Israel, is based on data and analysis done by our collaborators (Drs. J. Gonzalez, J. Martinez and W. Makalowski, this issue). They estimated the frequencies of 28 TEs (transposon elements) in fruit flies (*D. melanogaster*) from the ecologically tropic, hot, and dry south-facing slope (SFS) or “African” slope (AS) of EC and compared it with the TE frequencies on the temperate-cool and humid north-facing slope (NFS) or “European” slope (ES), separated, on average, by 250 m. The flies were sampled from two stations on each slope. We received their results, including the frequencies of each TE on each slope, and the probabilities of the statistical analyses (G-tests) of each TE separately. We continued the analysis of the inter-slope differences of the frequencies of the TEs, and based our different conclusions on that analysis and on the difference between micro (=EC) and macro (2000 km.) comparisons [Gonzalez et al. 2015 doi:10.1186/s13062-015-0075-4].

**Results:**

Our collaborators based all their conclusions on the non-significant results of each of the individual tests of the 28 TEs. We analysed also the distribution of the TE differences between the slopes, based on their results. Thirteen TEs were more frequent on the SFS, 11 were more frequent on the NFS, and four had equal frequencies. Because of the equalizing effect of the ongoing migration, only small and temporary differences between the slopes (0 – 0.06) were regarded by us as random fluctuations (drift). Three TEs were intermediate (0.08-0.09) and await additional research. The 11 TEs with large frequency differences (0.12 – 0.22) were regarded by us as putative adaptive TEs, because the equalizing power of ongoing migration will eliminate random large differences. Five of them were higher on the SFS and six were higher on the NFS. Gaps in the distribution of the differences distinguished between the large and small differences. The large gap among the 11 TEs favored on the NFS was *significant* and supports our rejection of drift as the only explanation of the distribution of the slope differences. The gaps in the distribution of the differences separated the putative TEs with strong enough selection from those TEs that couldn't overrule the migration.

The results are compared and contrasted with the directional effect of the frequencies of the same TEs in the study of global climatic comparisons across thousands of kilometers. From the 11 putative adaptive TEs in the local “Evolution Canyon,” six differentiate in the same direction as in the continental comparisons and four in the opposite direction. One TE, FBti0019144, differentiated in EC in *the same direction* as in Australia and in the *opposite direction* to that of North America.

**Conclusions:**

We presume that the major divergent evolutionary driving force at the local EC microsite is natural selection overruling gene flow. Therefore, after we rejected drift as an explanation of all the large slope differences, we regarded them as *putatively adaptive*. In order to substantiate the individual TE adaptation, we need to increase the sample sizes and reveal the significant adaptive TEs.

The comparison of *local* and *global* studies show only *partial* similarity in the adaptation of the TEs, because of the dryness of the ecologically tropical climate in EC, in contrast to the wet tropical climate in the global compared climates. Moreover, adaptation of a TE may be expressed only in part of the time and specific localities.

**Reviewers:**

Reviewed by Eugene Koonin, Limsoon Wong and Fyodor Kondrashov. For the full reviews, please go to the Reviewers’ comments section.

**Electronic supplementary material:**

The online version of this article (doi:10.1186/s13062-015-0074-5) contains supplementary material, which is available to authorized users.

## Background

The fruit flies at EC were collected by us, and the experiment was conducted by our collaborators, Drs. J. Gonzalez, J. Martinez and W. Makalowsky [1]. They sent the results and their conclusion to us, that all 28 tested TEs didn’t show adaptation because all separate G- tests of the frequency differences between the slopes, of each TE separately, were nonsignificant after the multi-comparison correction. We continued the analysis and took into account the influence of migration and the special fact that its adaptation depends only on the adaptation of the influenced locus, and not on the TE itself.

### “Evolution Canyon” model

**Fig. 1:**
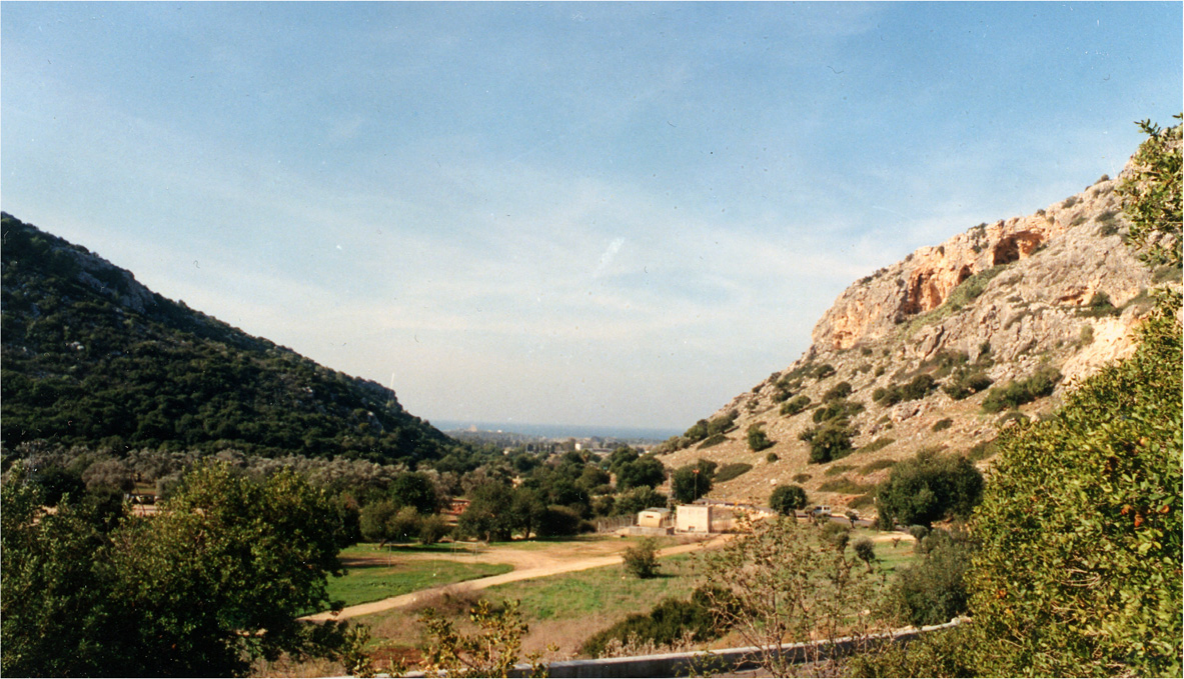
“Evolution Canyon” I, Mount Carmel: cross section. Note the forested "European slope" (NFS) and the abutting savanoid "African slope" (SFS)

**Fig. 2:**
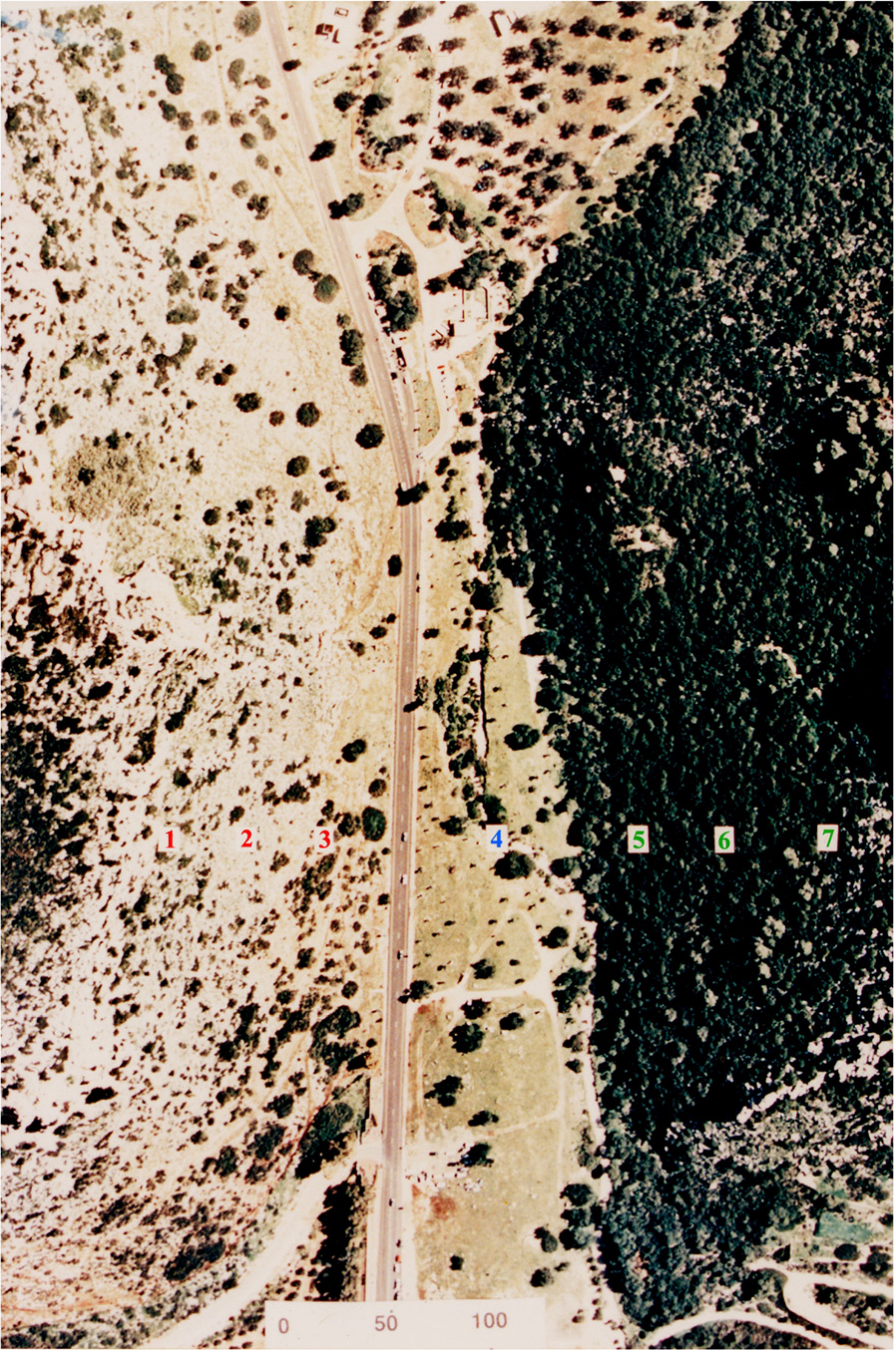
“Evolution Canyon” Mount Carmel: air view

**Fig. 3:**
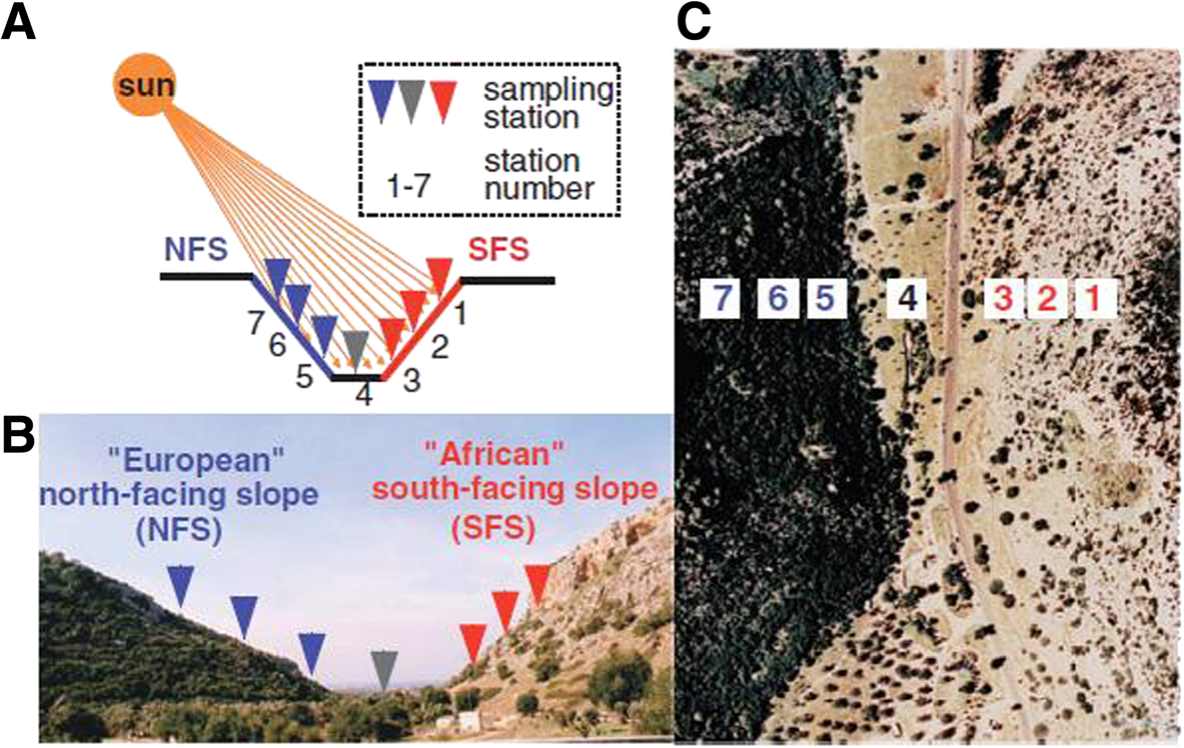
“Evolution Canyon” model in Israel. **a** represents the microclimatic model. **b** shows the cross section of the “Evolution Canyon (EC)”. **c** represents an air view of EC. The sharp divergence of savanna and forest habitats are seen in both the cross section of “Evolution Canyon” (EC) in (**b**), and its air view in (**c**). Collecting stations nos. 1 + 2 on the “African”, tropical, savannoid south - facing slope and stations 5 + 6 on the abutting “European”, north- facing slope are seen in both (**a**) and (**c**) [14]

**Fig. 4:**
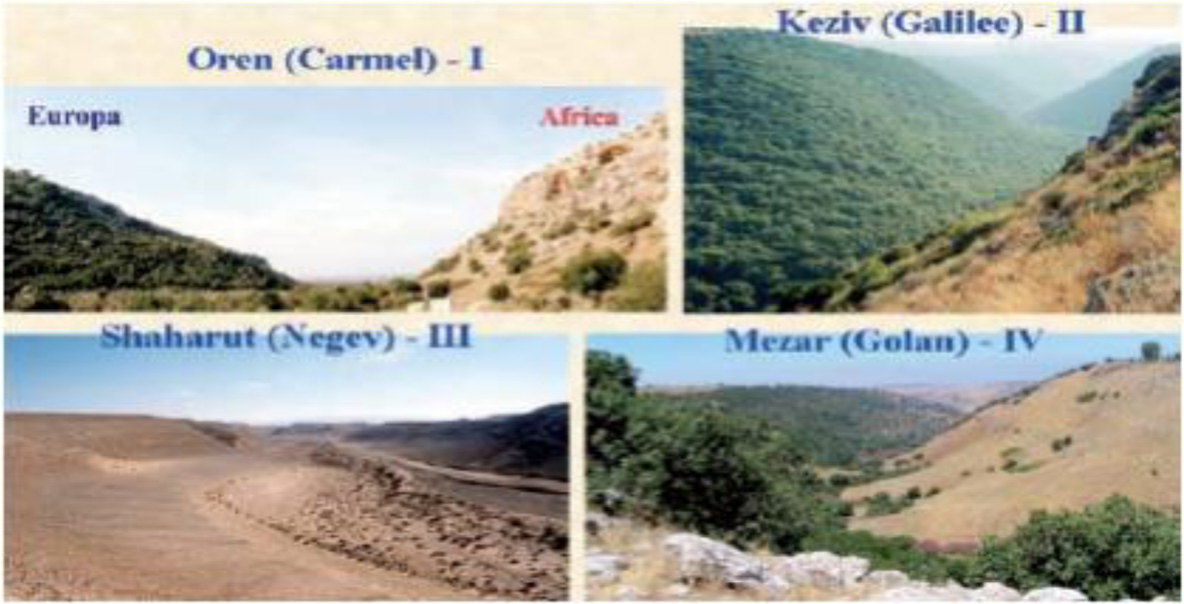
The four “Evolution Canyons” in Israel (EC **I** – **IV**, in the mountains of Carmel, Galilee, Negev, and Golan). Note the inter-slope divergence in vegetation, even at EC III in the Negev Desert (from [5]). The current paper deals with ECI in Mt. Carmel [5]

The “Evolution Canyon” (EC) model at Mt. Carmel (EC I) [2–7] (Figs. 1, 2, 3) is one of four “Evolution Canyons” (Fig. 4) studied in Israel representing microscale natural laboratories unfolding *evolution in action across life from bacteria to mammals*. The model explores biodiversity evolution, *inter-slope adaptive divergence,* and *incipient sympatric ecological speciation across life from bacteria to mammals* [2–7] (and Nevo’s list of “Evolution Canyon” publications at http://evolution.haifa.ac.il). The four ECs, I-IV (Fig. 4) are located in Israel in the Carmel, Galilee, Negev, and Golan mountains, respectively.

The canyons’ abutting opposite slopes are dramatically divergent ecologically due to microclimatic differences, despite the short distance, averaging 250 m in EC I at lower Nahal Oren, Mount Carmel. In EC I, 2,500 species were identified involving bacteria, fungi, plants, and animals in an area of 7,000 square meters. The “African slope” (AS), which is an ecologically tropical, savannoid, warm, and dry south-facing slope (SFS), is the more stressful slope due to higher solar radiation [8]. On this slope we identified higher biodiversity of terrestrial species than on the “European” (ES) north-facing slope (NFS), which is ecologically temperate, forested, cooler, and wetter. The AS is *water*-stressed and the ES is *light*-stressed [7]. In a long-term research program at EC I (1990- to date), we studied inter-disciplinarily life’s active evolution and the twin evolutionary processes of adaptation and speciation.

The EC microsite model is optimal for studying inter-slope biodiversity evolution, adaptive complexes [5], and incipient sympatric ecological speciation across life from viruses and bacteria through plants, fungi, and animals up to mammals [4, 9].

*Phenotypically*, soil fungi at the AS represent higher adaptive sexual reproduction, melanism, and robust conidia than at the ES; as well as xeromorphic plant phenotypes, thermotolerance, and drought tolerance in animals such as *Drosophila* and the spiny mice *Acomys* [2]. *Genotypically,* in 9 out of 14 (64 %) model organisms across life, we identified higher protein and DNA genetic polymorphism on the more stressful AS [4, 5]. Likewise, some model species on the AS exhibited higher mutation rate, gene conversion, recombination, DNA repair, larger genome size, different retrotransposons, and wide-genome gene expression positively associated with the higher stress of solar radiation, temperature, and drought on the AS [2–8, 10, 11]. Major adaptive complexes on the opposite slopes relate to high solar radiation, temperature, drought on the AS and the opposite stresses, i.e., lower solar radiation, cooler temperature and humidity on the ES, i.e. light deprivation stress (shade) generating adaptive complexes associated with chlorophyll and photosynthesis [12, 13]. Differences in retrotransposon were also described for *Bare-1* in the species *Hordeum spontaneum* [14]. The copy number of *Bare-1* elements correlates with the environmental conditions: the higher and drier locations are in the canyon the larger the copy number of *Bare-1* is, having the maximum number at the SFS slope (the one with the most extreme conditions).

*Phenotypically* and *genotypically*, organisms differ dramatically on the close, opposite slopes due to microclimatic divergence [2–8, 10]. Inter-slope adaptive divergence and incipient sympatric ecological speciation have been described at EC I across phylogeny in soil bacteria [15]; wild barley [4, 16]; fruit-flies [17, 18], beetles [19] and spiny mice [20]. The EC was dubbed the “Israeli Galapagos” because it is a cradle of origin for new species from bacteria to mammals through the process of incipient sympatric ecological speciation [4, 9] (see reviews on all of these aspects in [2–7, 9, 10]).

### *Drosophila melanogaster* at “Evolution Canyon”

Numerous studies have been conducted at EC I on *Drosophila melanogaster*, a cosmopolitan drosophilid out of the 9 drosopholid species found in EC I [21]. These studies demonstrated both adaptive complexes to the opposite slopes [11] and *incipient sympatric ecological speciation* between the slopes based on diverse biological perspectives. These include: morphology, physiology, behavior, genetics, genomics, natural history, demography, and inter-slope migration [9, 11, 17, 18, 22–37]. Hubner et al. [36] described whole genome differentiation of *Drosophila melanogaster* from EC I. A total of 572 genes were significantly different in allele frequency between the slopes; 106 of which were associated with 74 significantly over-represented in gene ontology (GO) terms, particularly related to stress, development, and reproductive success, thus corroborating previous observations of interslope divergence in stress response, life history, and mating functions. There were at least 37 “chromosomal” islands of interslope divergence and low sequence polymorphism, plausible signatures of selective sweeps, more so in ES flies. In a *D. melanogaster* repeatome paper [37], interslope populations exhibit a significant inter-slope difference in the contents and distribution of mobile elements and microsatelites. In summary, natural selection generated a unique case at the EC I microsite, of numerous adaptive complexes to the opposite slopes, as well as incipient sympatric ecological speciation at a microscale of this cosmopolitan species [9, 17, 18]. These adaptation and sympatric speciation phenomena proceeded in the face of ongoing distinct gene flow between the opposite slopes [33].

Global studies of a genome-wide screen for recent TE-induced adaptation in *Drosophila* identified 13 TE insertions likely to be adaptive [38, 39]. A substantial proportion of the adaptive TEs show population differentiation between north and south Australia and between south and north of the USA suggesting that these TEs are involved in adaptation to the temperate environments. In a review [40] 24 TEs were regarded as adaptive.

In the current work we tested if TEs previously identified in the *global* study [38, 39] as showing population differentiation patterns across ~ 2.000 km also show the same pattern at a microscale local level of 250 m (i.e., in an 8,000 times shorter transect from a *dry* ecological tropic (AS or SFS) to temperate domain (ES or NFS)) at ECI [2–7].

There are four main differences between our *micro* (local) study and the *macro* (global) continental comparisons. 1. In EC the ecological tropical *micro* climate at SFS (=AS) is of a *dry savanna type* [8] while the tropical climate in North Australia and in Florida, located in the south of North America, *have much more rainfall* [38, 39]. 2. In our current study the slopes have equal rainfall (but not equal humidity), while the mesic and tropical climates in the studies of Gonzalez et al. [38, 39] differ both in temperature and rainfall. 3. In our current study the results are the function of both selection and migration, which is possible by the small distance of 250 m [33]. In Gonzalez et al. [38, 39] the thousands of kilometers separating the fruit-fly populations eliminate any possibility of equalizing by migration. Although in EC selection overrules migration [6, 33], we expect that the observed migration [33] will reduce the differences in frequencies between the slopes, created by natural selection. 4. Genetic drift can even produce large and constant divergence in continental studies, but can’t do so in *micro* comparisons. Therefore, in the *micro* EC study, any large *inter-slope* difference is proof of selection. Differences caused by random processes will disappear, or become small, by the equalizing power of migration (and mating). Therefore, large differences existing between the slopes will demonstrate sufficiently strong selection; otherwise they will disappear. In sum, we expect that only part of the *locally* tested TEs will show the same pattern as the *globally* tested TEs, due to the aforementioned reasons.

## Methods

Flies were collected at EC I by bottle traps with banana bait as described in earlier studies [11].

The TE analysis was conducted by our collaborators, Drs. Jose Martinez, Josefa Gonzalez and Wojciech Makalowski [1] (published here, in this issue) on 4 populations: two from the warm-dry ecological tropical AS (stations 1 + 2) and two from the cool-humid temperate ES (stations 5 + 6), see details of population distribution in [41].

### Inversion frequency estimation in the four populations analyzed

The presence of cosmopolitan inversions In(2 L)t, In(3R)Payne and In(3 L)Payne was checked in all strains analyzed at EC I by Martinez and Gonzalez [1] using the following primers: for inversion In(2 L)t the primers described in [42] were used; for inversion In(3R)Payne the primers described in [43] were used, finally, for inversion In(3 L)Payne, the distal breakpoint sequences described in [44] were used to design primers to check for the presence and absence of the inversion. Primer pair 59-CCGGATGGACCACATAGAAC-39 and 59-CATTCTGGGCCTTATCATCT- 39 amplified the standard, but not the inverted chromosome, and primer pair 59- CCGCAAACGAACACTTA-39 and 59- GATTATGGACC- TAATGAAAGC-39 amplified the inverted, but not the standard chromosome [39]. Each TE located in an inversion is eliminated from the frequency calculation of that TE, which causes variation in the sample size of different TEs.

### TE frequency estimation

Martinez and Gonzalez checked whether each of the TEs analyzed in the *global* studies of [39, 40] was present and/or absent in the four *local* ECI populations, two on each slope (AS stations 1 + 2 and ES stations 5 + 6) using the PCR approach. The same primers were used to estimate the frequency of the TEs in other worldwide populations (Additional file 1: Table S1 in [38]). Briefly, for each TE, two primer pairs were designed: one was intended to assay for the presence of the TE, and the other was intended to assay for the absence of the TE. The pair that assays the presence of the TE consists of a Left (L) primer located within the TE sequence and the Right (R) primer located in the flanking region to the right of the TE insertion. The pair that assays for the absence of the TE consists of a Flank (FL) primer, which is located in the left flanking region of the TE and the R primer mentioned above (see in [38] for further details).

## Results

### Data sets of TEs

The frequency of a set of 18 putatively adaptive and 10 putatively neutral TEs, (previously described in *D. melanogaster,* from Australia and North America, in [39]) was estimated by our collaborators, Gonzalez et al., see in this issue [1], in four EC I populations: two populations collected in the “European” (ES) = North-Facing Slope (NFS), stations 5 and 6, and two populations collected in the “African” (AS) = South-Facing Slope (SFS) stations 1 and 2 (see Additional file 1: Table S1). First, the existence of the polymorphic chromosomal inversions, previously described in some *D. melanogaster* populations [39], was checked in the “Evolution Canyon” (EC I) populations. Polymorphic inversions in *D. melanogaster* show global latitudinal patterns [45]. To avoid the confounding effects of inversions on TE frequency estimates, (only for those TEs located inside chromosomal inversions), the strains that contained those particular inversions were removed before estimating their frequency. Three of the four chromosomal inversions described in *D. melanogaster* can be scored by PCR (see Methods). Overall, the frequency of inversions In(3R)Payne and In(3 L)Payne is low both in the ES (= NFS) and the AS (=SFS) populations (0.05 % and 11 %, respectively, for both inversions; (Additional file 1: Table S1). Inversion In(2 L)t is present in 30 % of the NFS and in 30 % of SFS strains analyzed.

Because the TE frequencies were not significantly different *within* slopes, we and our collaborators analyzed the data of the two populations from the same slope together (Additional file 2: Table S2). According to González et al. [1] two putatively adaptive TEs, FBti0019624 and FBti0020046, and one putative neutral TE, FBti0018879 showed significant inter-slope population differences at EC I (Table 1). However, none of these showed significant differences after applying the false discovery rate of multiple comparisons. Therefore, we can’t draw any general conclusion on the adaptation of the TE’s in general. The adaptation of each TE depends on the adaptation of the locus or DNA that it regulates. The TE will be adaptive if it increases the expression of a positively selected locus or decreases the expression of a locus that reduces the fitness of the organism. A clear example of an adaptive TE appears in [46]. The promoter region of *hsp70Ba*, a major inducible heat shock protein in *D. melanogaster*, was polymorphic for the P-element insertion and was 28 times more frequent on the NFS than on the SFS. The insertion was associated with decreased Hsp70 expression and a lower heat shock survival, which is not vital on the NFS but is important on the much more heat stressful SFS. Likewise, it also caused a reduction of the reproductive success of the fly. Therefore, the inactivation of the locus by the TE also caused an increase in reproductive success of the fly under the mesic conditions of the ES = NFS. Consequently, the *low* frequency of this TE is adaptive in warm climate, while its *high* frequency is adaptive in the cool climate.

**Table 1:** Frequency estimates of the 18 TEs belonging to adaptive families, and 10 TEs belonging to neutral families (based on [39]) in “Evolution Canyon” populations

			“Evolution Canyon” populations		
Flybase ID	Family^c^	Clinal patterns	NFS5^a^ & NFS6	SFS1^b^ & SFS2	*P* value^d^
FBti0018880	Bari1	-	0.70	0.69	0.9366
FBti0019056	pogo	AU08	0.84	0.79	0.5178
FBti0019065	pogo	-	0.76	0.73	0.7272
FBti0019144	Rt1b	NA	0.21	0.06	0.0802
FBti0019164	X-element	AU08	0.39	0.58	0.1293
FBti0019170	F-element	-	0.38	0.38	0.9250
FBti0019372	S-element	AU08	0.25	0.37	0.2346
FBti0019386	invader4	AU08, NA	0.48	0.32	0.1340
FBti0019430	Doc	-	0.98	0.98	0.8514
FBti0019443	Rt1b	AU07, AU08	0.35	0.44	0.3695
FBti0019624	hopper	-	0.75	0.54	**0.0351**
FBti0019627	pogo	NA	0.66	0.48	0.0973
FBti0019679	1731	-	0.89	0.87	0.7901
FBti0019747	F-element	-	0.15	0.21	0.4474
FBti0020042	jockey	-	0.31	0.32	0.9035
FBti0020046	Doc	NA	0.21	0.43	**0.0330**
FBti0020091	Rt1a	-	0.87	0.93	0.3342
FBti0020119	S-element	AU08, NA	0.34	0.34	0.9909
FBti0018879	BS	-	0.86	0.65	**0.0252**
FBti0019079	BS	NA	0.00	0.08	0.3913
FBti0019133	BS	-	0.69	0.89	0.0540
FBti0019165	BS	-	0.43	0.58	0.1431
FBti0019604	BS	-	0.33	0.34	0.8807
FBti0019771	1360	NA	0.40	0.40	0.9683
FBti0020056	BS	-	0.03	0.07	0.3674
FBti0020057	BS	-	0.65	0.48	0.1364
FBti0020125	BS	NA	0.53	0.50	0.7993
FBti0020155	1360	-	0.63	0.71	0.3810

^a^NFS = North-Facing Slope (=“European” Slope, ES)

^b^SFS = South-Facing Slope (=“African” Slope, AS)

^c^The adaptive families above the space and below the space the neutral families

^d^Significant values, p = < 0.05, are bold

### Inter-slope divergence

**Fig. 5:**
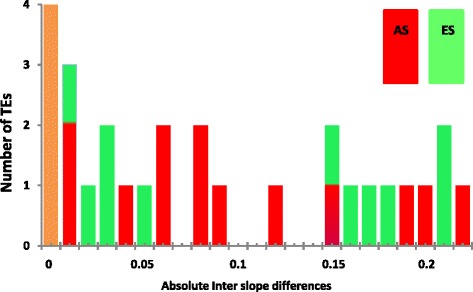
Distribution of the absolute values of the inter-slope differences between the frequencies of the TEs on each slope. For example, the first TE has a frequency of 0.32 on the AS and 0.48 on the ES. The difference is 0.32 – 0.48 = −0.16. A bar is entered near the value 0.16 in the figure. The colors mean in which slope is this TE more frequent (see in Fig. 6). Brown means equal frequency on both slopes

**Fig. 6:**
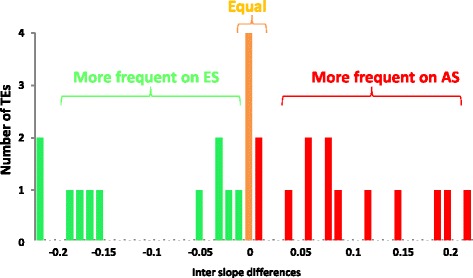
The distribution of the TEs inter-slope differences. Entries of each TE are marked by a bar above the value of the difference. All entries that are higher on ES are marked with a minus. For example, the first TE has a frequency of 0.32 on the AS and of 0.48 on the ES. The difference is 0.32 – 0.48 = −0.16. A bar was entered above the value of −0.16 in the figure. The colours mean in which slope is this TE more frequent (as in Fig. 5). Brown means equal frequency on both slopes

There are several very intriguing differences in TE frequencies between the slopes. There are 13 TEs higher on the AS (= SFS), and 11 TEs are higher on the ES (=NFS). In 4 TEs the inter-slope differences were less than 1 %, and we regarded them as having the same frequency on both slopes [Table 1 and Figs. 5 (of the absolute value of the differences) and 6 (of the directional values of the differences)]. The distribution of the values of the differences between the frequencies on the slopes is *not as expected in a random process*. The small inter-slope differences, 0–0.06 (Fig. 5), behave as expected from random fluctuations, i. e., higher frequency of the smallest differences (including the zero) and gradual lower frequency of the larger differences. The large inter-slope differences are between the absolute values 0.12 and 0.22. In contrast, their distributon is flat and even. They are dense at the highest values. Gaps separate the small from the large inter-slope differences (Figs. 5 and 6). In the left side of Fig. 6, where the TEs are “favored” on the ES (negative differences in Fig. 6), there was a large gap between the small and large inter-slope differences. In the gap not a single TE occurs with a difference between −0.06 and −0.14, while among the TEs “favored” on the AS (positive differences in the figures) the large gap is broken into 2 smaller gaps, by three TEs with intermediate inter-slope differences (0.08-0.09). Only with additional research can we estimate if those 3 TEs belong to small or large differences, i.e., have overruled the equalizing pressure, or are on the way to be equalized. The second reviewer kindly suggested a statistical test of the gap. He suggested to assume a flat distribution and equal sizes of the 3 regions, i.e., in that case there is a probability of a third to be in any region. If we test this gap (one of the regions of the tested TEs) than, by chance, each TE has the probability of 2/3 to be outside the gap and 1/3 to be inside the gap.

In Fig. 6 it is obvious that only in the left side of the distribution, of the 11 TEs “favored” on the ES, the gap is more then a third of the distribution. Therefore, we tested only that large gap and considered the similarity of the distribution among the TEs “favored” on the AS as supportive evidence.

The hypothesis of the flat distribution is nearer to the observed results (6 large + 5 small) than any other possible hypothesis of the distribution caused by drift, i.e., highest at the smallest differences and lower occurrence of the larger differences (nearer to “half” normal distribution). Therefore, if the flat hypothesis is rejected, all other possible hypotheses will be rejected also. We will take the 11 TEs which are higher on the ES (see in Fig. 6) and test the large gap between the two group of differences. No TE entered the gap. The probability that it happened by chance (drift) is (2/3)^11^ = 0.0116. Therefore, we can reject *significantly* the hypothesis that only drift or any random process created that gap. Thus, the *significant* gap separates between the group of small (0–0.06) and the group of large (0.12 - 0.22) differences of the TEs that are higher in the ES. In the right side of Fig. 6 the distribution of the TEs is similar to the significant distribution we described, only the gap is smaller and the 3 intermediates complicate the calculations. Therfore, we regard the 11 TEs, with the large inter-slope differences of the absolute value of 0.12 or more, as large, and presumably as a putative adaptive difference and not as the result of only random fluctuations. We assume they are selected for (or against) by the conditions in one of the slopes, at least. The 13 TEs that are higher on the AS show a similar weaker pattern. In the introduction we explained why in EC no large and constant inter-slope difference of TEs frequency are expected without selection, because of the existence of ongoing homogenizing widespread inter-slope migration. The 11 putative selective TEs consist of five TEs that are “favored” on the AS (FBti numbers 20046, 19133, 19164, 19165, and 19372) and six TEs that are “favored” on the ES (FBti numbers, 18879, 19624, 19627, 20057, 19386, and 19144). This could also be if they are selected against in the opposite slope instead of being “favored” in the more frequent slope, since we don’t know the frequencies of the TEs in the flies before they migrated to EC.

The fruit-flies first occupied the warmer AS, as their natural tropical habitat, and only afterwards moved from the AS to the ES, i.e., from the ecological tropical to the temperate microclimate and then adapted to the mesic climate [33]. Therefore, most of the TEs, which were “favored” on the AS, were selected *against* on the ES. The 3 intermediate TEs (Figs. 5 and 6) that differ by 0.08 or 0.09 between the slopes are all higher on the AS. The intermediate and large differences (0.12 to 0.22) have a flat distribution (ignoring the gaps), which is also unexpected for random fluctuations. Thus, this supports our suggestion considering the TEs with large differences (>0.12) as “putative adaptive”, the TEs with equal or small differences (= < 0.06) as “possibly caused by random fluctuation”, or neutral. The meaning of the 3 medium inter-slope differences must wait for future additional evidence. In the discussion we add an additional rejection of the hypothesis that all the large differences are created by drift. The rejection is based on the TE distributions among the stations in each of the slopes.

### Comparison of local EC pattern with global results

We compared the results of the 11 putative adaptive TEs with large inter-slope frequency differences, in our study, with their directional pattern in the global study of Australia and North America [39] (Table 2). From the 11 putative adaptive TEs, 6 TEs showed the same directional pattern of climatic change, in the *local* and *global* studies (FBti numbers 18879, 19165, 19386, 19624, 19627, 20057); four of the TEs showed an effect in the opposite direction (numbers 19133, 19164, 19372, 20046), and one TE (FBti0019144) cannot be classified because the change in EC is in the same direction as that found in Australia, but in the opposite direction to the change in North America (Table 2). The contrasting directional effects of the TE FBti0019164 could be explained because it regulates a locus that is responsible for survival of the fly during starvation. Starvation may be a temporary stress in different years of the same or different climates.

**Table 2:** Comparison of frequency differences of the eleven putatively adaptive and the three intermediate TEs, between the locally two abutting slopes of EC (detailes in Table 1), with the TE frequency differences between the mesic and tropical climates in Australia and in North America in *D. melanogaster* populations [39]

Flybase ID FBti00…	Family	Differences in:			Regulation of function^a^	EC	Australia 2007	Australia 2008	North America
		NFS	SFS	*P*-value					
…19144	Rt1b	0.21	0.06	0.0802	?	M 0.15@	M 0.26*	M 0.15@	T 0.27*
…19164	X-element	0.39	0.58	0.1293	Starvation	T 0.19	M 0.14	M 0.45***	M 0.16@
…19372	S-element	0.25	0.37	0.2346	Mitotic cyc.	T 0.12	M 0.24*	M 0.22**	M 0.14@
…19386	invader4	0.48	0.32	0.134	Meiotic cou.	M 0.16	M 0.20*	M 0.33***	M 0.30**
…19624	hopper	0.75	0.54	**0.035**	?	M 0.21*	T 0.12	M 0.22*	T 0.03
…19627	pogo	0.66	0.48	0.0973	Mitotic cyc.	M 0.18@	M 0.07	T 0.02	M 0.26*
…20046	Doc	0.21	0.43	**0.033**	Immune	T 0.22*	M 0.04	M 0.12	M 0.25**
…18879	BS	0.86	0.65	**0.025**	?	M 0.21*	M 0.06	M 0.22*	M 0.26*
…19133	BS	0.69	0.89	0.054	?	T 0.20@	M 0.05	M 0.20@	M 0.08
…19165	BS	0.43	0.58	0.143	?	T 0.15	M 0..11	T 0.07	T 0.23*
…20057	BS	0.65	0.48	0.1364	?	M 0.17	M 0.12	M 0.17@	T 0.12
The TEs with intermediate differences:									
19443	Rt1b	0.35	0.44	0.3695	Circadian	T 0.09	M 0.34***	M0.33***	M 0.03
19079	B**S**	0.00	0.08	0.3913	?	T 0.08	M 0.06	M 0.22*	M 0.26*
20155	1360	0.63	0.71	0.3810	?	T 0.08	M 0.15	T 0.02	T 0.11

^a^The functions are selected from Table 3 in [39]

*Abbreviations:*

*M* Higher frequency in the mesic cooler micro climate, i.e., in the local north-facing slope (NFS = ES) of EC, or globally in South Australia or in northern North America

*T* Higher frequency in the ecologically tropic warmer microclimate, i.e., in the local south-facing slope (SFS = AS) of EC, or globally in North Australia or in southern North America (Florida)

? = Function of the regulated locus is unknown

Significance: @ = *p* < 0.1; * = *p* < 0.05; ** = *p* < 0.01; *** = *p* < 0.001

## Discussion

### Expectation of frequency differences in *local* and *global* studies

The expectations in the *local* study in EC are basically different from the expectations in global large- distance comparisons [38, 39]. If there is a large distance between the compared areas, the same difference in frequency can be caused by drift or by selection. In the local study at EC we don’t expect large differences without strong enough selection, because they will disappear by the equalizing power of the ongoing migration between the slopes [33]. We expect large differences only if selection overrules migration [6]. But in that case migration may only decrease the difference. Therefore, in the *local* (EC) study we may need a larger sample size than in the *global* comparison in order to reach significance.

### Inter-slope putative adaptive patterns

After the multiple comparison correction (= false discovery rate) was applied, no general conclusion about the adaptation of the TEs can be drawn. It is also not expected because the adaptation of any TE doesn’t depend on the TE itself. The adaptation of each TE depends mainly on the adaptation of the function of the locus (or loci or region of DNA) that it regulates and not on its own function. Therefore, we should partition the TEs according to their putative adaptation drawn from their frequencies. Those TEs that have large differences between the slopes *have putatively different fitness on each slope*, while those with no or small differences in frequency *putatively have small fitness differences or even equal fitness on both slopes.*

Our results indicate a similar amount of TEs with higher frequency on each of the opposite slopes (AS and ES). There are different trends of TE frequencies, presumably suggesting different *slope-specifc adaptations* due to natural selection on the function of the regulated DNA. This pattern was discovered because we analyzed the inter-slope *differences* regardless of the values per se (Table 1). These inter-slope differences appear in Fig. 5 (the absolute differences) and 6 (directional differences). The outcome is clearly a *non-random* distribution of the inter-slope differences in frequency, suggesting *slope-specific* putative adaptations: (i) six TEs with large higher values on ES and (ii) five TEs with large higher values on the AS. These two groups [(i) and (ii)] are putatively selected; because the ongoing migration [33] didn’t eliminate them, and (iii) 14 TEs showed small or no inter-slope differences, putatively with equal fitness (=“neutral”) or only weakly selected, so that the selection doesn’t overrule the migration. (iv) Three intermediate TEs need additional testing, which will be obtained by a larger sample size (Figs. 5 and 6).

In describing the results we ignored the previous partition of “adaptive” and “neutral” TEs [39]. From the 11 putatively selected TEs in EC, 4 are from the 10 defined as putative neutral in [38]. In EC a large difference can’t occur without natural selection and therefore we regarded them as putatively adaptive. That result may also support the possibility described in [39] that a TE from a “neutral” family can be selected and adaptive under some conditions.

Remarkably, if an overall statistical analysis is conducted, the aforementioned *meaningful inter-slope differences are not observed or ignored*. Likewise, there is no justification to ignore the nonsignificant differences because the function of each TE is different and each TE must be tested *independently*. In addition, the power of the test is not calculated, but only if that power is strong enough can we reveal the meaning of *non-significant*. From the results we can conclude that the test is *too weak* to find the significance of real differences that are smaller than 0.2. This pattern of large and small *inter-slope differences in genetic markers* (allozymes and DNA) has been identified in earlier studies (Nevo “Evolution Canyon” list at http://evolution.haifa.ac.il).

### Global versus local studies

Notably, out of the 13 putative adaptive TEs in the *global* study (Australian and American comparisons [39]), four TEs (31 %) appear also to be *locally* putatively adaptive at EC I. Likewise, out of the 18 analyzed TEs, which belong to adaptive families used in that global study, 11 (61 %) are putatively neutral in EC. This indicates that the similarity between *global* and *local* studies may be only *partial*, since the selective regime and climate differ, as explained earlier. In *global* studies the tropics are *wet* and hot (Australia and North America) whereas at the *local* study (at EC I), the ecologically tropical AS is *dry* and hot. The four main differences found in the *micro*-comparison between the EC and findings in the global studies of Gonzalez et al. [38, 39] are described at the end of the Introduction. These differences include (1) *dry* versus *wet* tropical climate, (2) migration exists only in the *micro* or *local* inter-slope comparison, (3) drift can’t produce a *locally* large inter-slope divergence of frequencies, and (4) locally equal rainfall on the opposite slopes (but not humidity) in the EC. Moreover, in a microsite research, such as at EC, even if selection overrules migration [6], the weaker gene flow will decrease the differences in frequencies between the slopes created by the stronger selection. Therefore, in EC a larger sample size is needed in order to achieve the same significance, as in the global study. If there is large geographical separation between the two compared climates, as in Australia and North America (TE studies of Gonzalez et al. [38, 39]), the separation will allow random drift to increase differences. Therefore, the expectation to get the same pattern of adaptation of the same TEs in *local* as in *global* comparisons is only *partially* correct. By comparing frequencies on the opposite slopes at EC, we identified different presumably putatively adaptive TEs, prevailing on each of the slopes generated by natural selection, typical to the mesic (ES) versus xeric (AS) local slopes.

### Differences between the two papers on the same experiment

A second paper based on the same experiment, published back-to-back in this issue by Gonzalez et al. [1], didn’t find population differentiation of TEs in EC I [1]. There are a few reasons for the different conclusions. 1. The authors regard the 28 tests of adaptation for each TE as repetitions. But, as explained earlier, the adaptation of each TE depends not on the TE itself but on the adaptation of the locus it regulates. Each TE regulates different locus (or loci?), and therefore the test of each TE is an independent test and not a repetition. Therefore, the correction of multiple comparisons must be done differently. They also regard equally the 10 neutral TEs from neutral families and the 18 “adaptive” TEs from adaptive families. Therefore, the correction of 28 repetitions is an over-correction. Previously, we explained that the influence of the TE is by causing a mutation or by the regulation of the neighboring locus (or loci) in other ways. Therefore, the tested hypothesis should be “is this specific TE adaptive” and not that the “TEs are collectively adaptive”. Moreover, we see three types of TEs: “favored” in AS, “favored” in ES, and neutral. It seems that it depends on the identity and function of the influenced loci in a specific environment. The pattern can be tested in the future at EC II in western Upper Galilee which is a similar, though not identical, repetition of ECI. 2. In the *local* EC, in contrast to the *global* comparison, genetic drift can’t create large and consistent differences. Therefore, observed large differences, even if *non-significant*, represent weaker proof of selection, or any other driving force*, but not genetic drift or randomness*. 3. We regard non-significant differences differently. In Gonzalez et al. ([1], this issue) they regard a non-significant difference as no difference at all. We regard it as an observed difference, which has not been proven yet, and needs additional research or additional support, as by increasing the sample size, in order to achieve significance or it will continue to be an unproven observation. In the analysis of Gonzalez et al. [1] the probability wasn’t corrected by the equalizing power of the existing migration. Our conclusions were drawn, instead, from the unusual distribution of the differences (Figs. 5 and 6).

There are no general conclusions for the general adaptation of all TEs because the number of putatively adaptive TEs favored on the AS are similar to the number of putative adaptive TEs favored on the ES and, therefore, we didn’t recognize any overall deviation in favor of one slope. Importantly, in a recent review of population genomics of transposable elements in *Drosophila* [47], it was concluded that although most of TE insertions are deleterious or neutral, some TE insertions increase the fitness of the individual that carries them and play a role in genome adaptation.

### Significant rejection of the hypothesis of the random (drift) distribution of the TEs with large slope differences

In the results we divided the 28 TEs into: one group of 11 TEs with large differences in frequencies between the slopes, which we considered as putatively adaptive caused by natural selection, and to a second group of 14 TEs, which have small or no differences at all between the slopes, which may be caused by drift and weak selection and will be also temporary. Three uncertain TEs with intermediate differences are left between the groups and are waiting for additional research. The gap between the groups is larger and clearer between the TEs that are more frequent on the European slope. In Table 3 the frequency of each TE on each slope is divided into the frequencies in each measured station. In each slope there are two stations. The sums of ranking of the TE frequencies for each slope are given. There are 6 possible results of 3 types: 7–3, 6–4, 5–5 (2 + 3 and 1 + 4), and vice versa. If we assume randomness (drift) we have equal probabilities to receive any of those six possibilities. Therefore, under the hypothesis of randomness, we expect that the probability of each possibility is 1/6 and each type is 1/3. The results in Table 3: the 11 putatively selective TEs, the large differences, have seven 7–3, three 6–4 and one intermediate 6.5-3.5. Not a single 5–5 occurs in this group. The probability that 5–5 will not occur in a single TE is 2/3. That in 11 TEs, we will not get a single “5-5” by chance, has the probability of (2/3)^11^ = 0.01156. This means that we can reject significantly the hypothesis of randomness with more than 95 % confidence on the base of the distribution of the frequencies of the putative adaptive TEs among the stations. The conclusion is that at least part of those TE frequency differences between the slopes are not created by chance and are probably the result of natural selection.

**Table 3:** Rating of TE frequencies in the stations on each slope of “Evolution Canyon”, Carmel Mountains, Israel

	Group selective? Large diff.	NFS		St.6		St.5		SFS		St.2		St.1		Sum of rating of st.		Difference between slopes
		Frequency	N	Freq	N	Frequency	N	Frequency	N	Frequency	N	Frequency	N	on NFS	on SFS	
A	FBti0019386	0.48	20	0.46	14	0.5	6	0.32	25	0.25	16	0.44	9	7	3	0.16
A	FBti0019624	0.75	20	0.79	14	0.67	6	0.54	26	0.50	16	0.60	10	7	3	0.21
A	FBti0019627	0.66	19	0.61	14	0.8	5	0.48	23	0.40	15	0.63	8	6	4	0.18
A	FBti0019144	0.21	14	0.25	10	0.13	4	0.06	16	0.11	9	0.00	7	7	3	0.15
N	FBti0018879	0.86	18	0.85	13	0.9	5	0.65	26	0.53	16	0.85	10	6.5	3.5	0.21
N	FBti0020057	0.65	17	0.68	11	0.58	6	0.48	20	0.46	14	0.50	6	7	3	0.17
A	FBti0019372	0.25	20	0.25	14	0.25	6	0.37	26	0.44	16	0.25	10	4	6	0.12
A	FBti0019164	0.39	14	0.45	10	0.25	4	0.58	18	0.59	11	0.57	7	3	7	0.19
A	FBti0020046	0.21	17	0.18	11	0.25	6	0.43	22	0.43	14	0.44	8	3	7	0.22
N	FBti0019133	0.69	13	0.72	9	0.63	4	0.89	18	0.86	11	0.94	7	3	7	0.2
N	FBti0019165	0.43	20	0.54	14	0.17	6	0.58	25	0.63	15	0.50	10	4	6	0.15
	?															
	Intermediate	diff.														
A	FBti0019443	0.35	20	0.36	14	0.33	6	0.44	26	0.44	16	0.45	10	3	7	0.09
N	FBti0019079	0.00	20	0.00	14	0	6	0.08	26	0.13	16	0.00	10	4	6	0.08
N	FBti0020155	0.63	20	0.64	14	0.58	6	0.71	26	0.81	16	0.55	10	5	5	0.08
	Temporary															
	Small diff.															
A	FBti0019679	0.89	18	0.85	13	1	5	0.87	23	0.89	14	0.83	9	6	4	0.02
A	FBti0019065	0.76	19	0.73	13	0.83	6	0.73	26	0.81	16	0.60	10	6	4	0.03
A	FBti0018880	0.70	20	0.71	14	0.67	6	0.69	26	0.69	16	0.70	10	5	5	0.01
N	FBti0020125	0.53	18	0.54	12	0.5	6	0.50	25	0.50	16	0.50	9	6	4	0.03
A	FBti0019056	0.84	19	0.81	13	0.92	6	0.79	26	0.75	16	0.85	10	6	4	0.05
A	FBti0020091	0.87	19	0.92	13	0.75	6	0.93	22	1.00	14	0.81	8	4	6	0.06
N	FBti0020056	0.03	19	0.00	13	0.08	6	0.07	22	0.07	14	0.06	8	5	5	0.04
A	FBti0019747	0.15	20	0.14	14	0.17	6	0.21	26	0.19	16	0.25	10	3	7	0.06
A	FBti0020042	0.31	18	0.29	12	0.33	6	0.32	22	0.36	14	0.25	8	5	5	0.01
N	FBti0019604	0.33	20	0.32	14	0.33	6	0.34	25	0.34	16	0.33	9	3.5	6.5	0.01
	Zero(<0.01)															
A	FBti0019430	0.98	20	0.96	14	1	6	0.98	26	1.00	16	0.95	10	5.5	4.5	0
A	FBti0019170	0.38	20	0.39	14	0.33	6	0.38	26	0.38	16	0.40	10	4	6	0
N	FBti0019771	0.40	20	0.43	14	0.33	6	0.40	24	0.37	15	0.44	9	4	6	0
A	FBti0020119	0.34	19	0.27	13	0.5	6	0.34	22	0.39	14	0.25	8	6	4	0

Stations 5 and 6 on the ES = NFS and stations 1 and 2 on the AS = SFS

*Abbreviations:* *A* From an adaptive family (39), *N(only in 1* ^*st*^ *column)* From a neutral family (39), ? - Putative or unknown, st. = Station; N (in header) = Sample size; diff. = Inter-slope difference of TE frequency

### Extensive whole genome TE study at EC

In a second extensive study of TEs of *Drosophila melanogaster* in EC [37], instead of estimating frequencies of each TE, the number of different types of TEs on both slopes was counted and compared. Significantly more retrotransposon elements were found on the ES than on the AS. This was found separately for LTR and non-LTR retroTEs. The differences involved hundreds of TEs for each class. In the TIR class of TEs a much smaller non-significant difference was found. This second comprehensive study of the *Drosophila melanogaster* repeatome at EC I [37] showed that flies from the ES carried about 5 % more transposable elements than those from the AS, in parallel to a suite of other genetic and phenotypic differences between ES and AS in *D. melanogaster* and other model organisms. The location of nearly 50 % of all mobile element insertions were *slope-unique*, with many of them disrupting coding sequences of genes critical for *cognition, olfaction*, and *thermotolerance,* which are consistent with other adaptive complexes and incipient sympatric ecological speciation of *D. melanogaster* on the opposite slopes.

From this study and all other studies in EC (Nevo “Evolution Canyon” list, September 2015 in http://evolution.haifa.ac.il), some reviewed in the Introduction, we can conclude that **“**Evolution Canyon” (EC) is an optimal microsite natural laboratory for studying *evolution in action across life* from viruses and bacteria through plants, fungi, and animals, both invertebrates and vertebrates. Studies conducted in this long-term project initiated in 1990 include biodiversity evolution, divergent ecological adaptive complexes to local microclimatic divergence, and the most revealing, *incipient sympatric ecological speciation across life* [9]. All these studies make EC an optimal evolutionary laboratory [2–11, 17–37, 48]. All studies to date, including TEs, showed ecological-genetic and ecological genomic adaptive divergences, both *phenotypically* and *genotypically*, in accordance with the inter-slope microclimatic regimes at a small distance of 250 m, driven by natural selection, and natural genetic engineering [49].

## Conclusions

The present study of TEs clearly indicates inter-slope divergence between the micro-climatically divergent slopes with ongoing gene flow. In the EC only if there is a strong enough power, as of natural selection, to overrule the equalizing pressure by the migration, can a stable and large divergence be observed. Therefore, this conclusion is based on the existing inter-slope migration which didn’t allow drift to create stable and large differences. We also found that the distribution of the slope differences negate genetic drift as a general explanation of the results.

In order to find which of the 11 putatively adaptive TEs are really adaptive, we must achieve significance for each individual TE. In order to reach that goal we must continue the research and increase the sample size, as explained earlier. Moreover, to learn more about the generality of our results, we must wait until the ongoing TE reaserch deciphers how the jumping of a TE to a new location (which happened in 50 % of the TEs in EC) [37] influences the identity of the locus (or loci) it regulates, which may change the adaptation of the TE.

This study was initiated in order to compare and contrast the *global* pattern of adaptation of the 28 TEs with the same TEs at the *local* EC. Only *partial* parallelism was found. The main reason is, most probably, that the tropical slope at the local EC is similar to a *dry savanna*, rather than to a *wet tropical climate, as exists* in global North America and Australia tropics. Also, in EC only a strong enough natural selection will overrule gene flow while in the global study, even a weak selection can create a large difference. Moreover, each TE regulates different functions, some are adaptive only in part of the time and localities, and we don’t expect complete parallelism.

Future planned genome and transcriptome studies and long-term transplant experiments of AS populations to the ES and vice versa, and individual (not pooled) genome and transcriptome studies, could highlight much of the remaining enigmatic perspectives of TE evolution of *D. melanogaster* at “Evolution Canyon”.

## Reviewers’ comments

Readers should note that this manuscript was submitted and published in parallel with Gonzalez et al., 2015 (*Biology Direct* 2015, doi:10.1186/s13062-015-0075-4). Although submitted together, both manuscripts were reviewed independently by the same three reviewers. Some comments within the reports below may refer to Gonzalez et al., 2015.

### Reviewer’s report 1

Eugene Koonin, NCBI, NLM, NIH, United States of America.

**Reviewer comments:** In this article that is appearing back to back with the article by Gonzalez et al., Beiles et al. address the same question, namely putative adaptive roles of TEs in the differentiation of Drosophila populations on the opposite slopes of the Evolutionary canyon on Mount Carmel. Beiles et al. investigate the same set of TEs as Gonzalez et al., and similarly fail to detect significant differences after the appropriate multiple test correction. However, they arrive to the opposite conclusion, on the basis of the observed moderate (not reaching statistical significance) differences. I do not seem to fully grasp the logic here. Beiles et al. indicate that the adaptive roles of TEs depend not simply on their frequencies but rather on the effects of the TEs on the expression of the specific adjacent genes. This is a fully plausible argument, I think this is indeed true. However, the argument misses the point which is not that these TEs are not adaptive; some of them well might be. The point is, simply, that the significant differences in the frequencies of 18 TEs observed between remote populations in America and Australia were not reproduced in the comparison of the populations from the two slopes of the EC. Thus, in this case, the appropriate null hypothesis, namely that the frequencies of the TEs are not significantly different and accordingly, there are no direct indications of their adaptive role on the microscale, could not be falsified.

### Reviewer’s report number 2

Limsoon Wong, NUS, Singapore.

**Reviewer comments:** This paper evaluates a set of 28 transposable elements (TEs) for adaptive divergence at “evolution canyon”. Methodologically, this paper has limited novelty. However, there are several interesting aspects in the analysis and discussion:

1. When evaluated individually, after adjusting for multiple testing, none of these shows significant difference in frequencies between the two locations.
2. The authors claimed that, in spite of this, a few of the TEs can be considered to have exhibited sufficiently large difference in frequencies between the two locations. A threshold of ~0.2 in frequency was used. However, it is unclear how such a threshold was determined and whether it should be regarded as a universal threshold that would also be valid in other studies.
3. The authors argued that the said TEs in 2/should be considered in their own right as they have their own functional roles. However, the authors did not state what precise functions these TEs have and how those functions are relevant for adaptation to the two locations. Thus I think this part of the argument is not so convincing.
4. The authors highlighted that the distribution of the frequencies of the 28 TEs shows a high proportion of TEs at the low-difference end and the high-difference end, but a very low proportion of TEs at the medium difference end. They claimed this bi-modal distribution is suggestive of TEs at the low-difference end being non-adaptive, and at the high-difference end being adaptive. I agree that this bi-modal distribution is significant. At my own rough calculation, assuming a uniform null distribution and the TEs are sufficiently far apart, a TE has a 1/3 chance to show a medium difference. Thus, there is a (2/3)^28 = 0.00001 chance of no TE showing a medium difference, which is rather significant. So I think there is some evidence for the authors’ claim.
5. However, there are two caveats. Firstly, the paper does not tell how many flies were measured in each location. A sample size that is not sufficiently large may not provide a sufficiently accurate estimate of the TE frequencies in the population at the two locations. Secondly, the 28 TEs are sufficiently far apart so that frequencies are effect of independent evolution.

**Authors’ response:***We want to thank both reviewers for agreeing to review our manuscript.*

*The first review, of Dr. Koonin, doesn’t regard our arguments for a basic difference between the micro and macro comparisons. In the macro comparison both selection and drift can create the difference. In the micro study the existing migration between the slopes, equalizes the different frequencies. Only strong enough selection can overcome the equalizing pressure. We draw the conclusion from the distribution of the differences of the frequencies between the slopes. As to the statistical difference (between low and high differences) the reviewer Prof. Wong estimated it and found that the dispersion is significant. We thank the second reviewer, Prof. Wong, who calculated this significance of the partition into two distributions. Chance alone is expected to create a single distribution with one intermediate pick. In the micro study, at EC, with migration, drift and selection behave differently. The small differences (0 – 0.06) can be created by drift and/or by weak selection, and are temporary, while the large differences (0.12 – 0.22) can be created only by strong enough selection that is able to overcome the equalizing pressure. Therefore, in the micro study chance or drift cannot be an explanatory model.*

*Dr. Koonin ignored also our claims against the multiple comparison correction. We admit that we cannot conclude any general conclusion about TE’s. The function of the TE is to regulate or mutate a (neighboring?) locus (or loci?). The TE will be adaptive, only if the locus influenced is adaptive. Therefore, each test of a TE is an independent test. Consequently, we concluded that we have no general conclusion about the adaptation of TE’s in general. We can only draw independent conclusions about the adaptation of each individual TE.*

*Dr. Koonin also expected that the same TE’s will be adaptive in both global (=macro) and local (=micro) comparisons. He ignored our argument that the ecologies differ in both comparisons. In the global comparison both climates are wet, while the warm slope of “Evolution Canyon” is much DRIER (=SAVANNOID) then the HUMID tropical climates in the global study. Therefore, we expect only partial similarity between the adaptive TE’s of both studies. Moreover, in the article we mentioned one TE, that is significantly adaptive in both continents,* i.e.*, Australia and America, but in the opposite directions.*

*In the second review Prof. Wong asked some questions.*

1. *In paragraph 2 he asked how “large” and “small” are decided? And if ~0.2 is a general border. Our decision is based on the distribution. The distribution of the small differences, from −0.05 to +0.06, of 14 TEs, is similar to a normal distribution, is nearly symmetrical, and has one central peak, as expected from a random variable. After the gaps there are 11 TEs with slope differences of absolute values 0.12 – 0.22 which can’t be produced by drift, following migration between the slopes. The large gap among the TEs with higher frequency on the ES separates between the “large” and “small” differences. No general meaning is attached to the 0.2 or 0.12.*
2. *In paragraph 3 Prof Wong asked about the function of the TE. The TE influences the locus in which it is located or that of neighboring loci. The TE may reduce or enhance the expression of a locus, or cause its mutation. This is the function of the TE. In the text we gave an example how a TE that silences a harmful locus is adaptive. In a larger study of D. melanogaster flies on TEs at EC (Kim et al., 2014 [*37*]), where thousands of mobile elements have been studied, 50 % of the insertions were ‘slope specific’, with many of them disrupting coding sequences of genes for cognition, olfaction, and thermoregulation, among others. Adaptation of a TE is not a general hypothesis about the TEs, but a hypothesis about the adaptation of the influenced locus.*
3. *In paragraph 5 Prof. Wong asked about the sample size and the locality of the TEs. The sample size is 20 strains of flies from the NFS and 22 strains from the SFS. This is taken from the attached supplementary Table*1*in the original response. I attached a supplementary table from the accompanied paper of Gonzales et al. which conducted our experiment and analyzed it differently.*

### Reviewer’s report number 3

Fyodor Kondrashov, Center for Genomic Regulation, Spain.

**Reviewer comments:** This is a more voluptuous manuscript that includes an introduction to the studies undertaken at the particular site and a more detailed description of examples of adaptive differences. Furthermore, the authors provide a different analysis and text from that of the back-to-back submission of Gonzalez and colleagues. Since the data has been generated by the authors I feel this manuscript should appear together with the co-submitted manuscript. It is an example of the two sets of authors obtaining the same result but then writing different words about it. What the present authors describe as their conclusions is strange at best and potentially greatly damaging to them and to the field. The authors acknowledge that there is very little (if any) statistical significance to suggest a difference in selection on the TEs between the two populations. Astonishingly they then proceed to say that the lack of a statistical difference, in their own words, should be regarded as “an observed difference, which has not been proven yet, and needs an additional experiment or additional support”. Without denying the authors the right to regard the observed (non)difference as anything they wish to believe, it must be noted that such statements are just bad science. The title of the manuscript becomes highly misleading, and the same thing applies to the abstract of the manuscript (for example the cut-off values reported in the abstract and the manuscript appear to be arbitrary). The approach taken by the authors, to proclaim conclusions based on belief rather than evidence, is particularly troubling given that the subject matter of selection and evolution is often the victim of such an approach from the creationist and intelligent design crowd. Given the spirit of *Biology Direct* as a journal that provides open peer review I believe it is possible to offer publication to the present manuscript, on two conditions. First, I believe that the co-submitted manuscript of Gonzalez et al. must also be accepted. Second, the authors must rewrite their title and abstract to provide a fairer description of the actual data.

**Authors’ response:***We want to thank the reviewer for agreeing to review our manuscript.*

*In the review Dr. Kondrashov doesn’t consider at all any of our arguments that cause the different conclusions that we draw from the same results. Our arguments are based on the basic differences between the micro (Evolution Canyon) study and the macro (North American and Australian) studies. In contrast to what is said in the review our dispute with our colleagues is based not on beliefs but on facts and observations. In addition to existence of migration (see later) they expect also similar results in Evolution Canyon as they got in their Australian and American researches. We discussed the large differences in climate (rainfall) and migration (distance) and therefore, we expect that the similarity of the adaptive TEs will be only partial and will be much weaker and therefore also need larger sample size.*

*In the macro comparisons both selection and drift can create the observed differences and the analysis tries to find out which difference is caused by adaptation and which one represents random changes (drift). In our present micro study the existing migration of Drosophila between the slopes (see Pavlicek et al.* [33]*), equalizes the different frequencies on the slopes. Only strong enough inter-slope selection can overcome the equalizing pressure. Therefore, random processes can’t create a large and constant difference in “Evolution Canyon”, except in an organism that do not migrate between the slopes. Therefore, any observed large and/or constant difference observed into “Evolution Canyon” must be the result of selection or any other force, but, not of drift. That has not been mentioned by the reviewer and our colleagues.*

*The problem is how to define small and large? We draw the conclusion from the distribution of the differences of the frequencies between the slopes. It is obvious that the small ones are concentrated near zero, from −0.06 to 0.05, while the large ones are concentrated into two equal, flat and dense groups, of the absolute values 0.12 – 0.22. One is selected for on the NFS and the other is selected against on the NFS or oppositely on SFS. We added a statistical test of the partitioning between the two groups: The large and the small. We thank the second reviewer, Prof. Wong, who calculated the significance of the partition into two distributions and found the partitioning significant. Chance alone is expected to create a single distribution with one intermediate pick. In the micro study, at EC, with migration, drift and selection behave differently. We hypothesize that this observed distribution separate between the random and the selected TEs. The small differences (14 TEs between −0.05 and + 0.06) can be created by drift and/or by weak selection, and are temporary. Their distribution looks as expected, similar to a normal distribution,* i.e.*, is nearly symmetrical, and has one central peak. The two groups of large differences are flat and far from normality. In EC large difference between the slopes can be created only by strong enough selection that is able to overcome the equalizing pressure. Therefore, in the micro study chance or drift cannot be an explanatory model.*

*The reviewer ignored also our claims against the multiple comparison correction. We admit that we cannot conclude any general conclusion about TE’s. The tested hypothesis is not about the adaptiveness of TEs, but, only on the adaptiveness of a specific individual TE in this location at that time. The function of the TE is to regulate or mutate a locus (that it is inserted in it, a neighboring one or more loci). The TE will be adaptive, only if the locus that is influenced by it, is adaptive. If the TE will change its location it will influence other loci. In a larger study of D. melanogaster flies on TEs at EC (Kim et al., 2014 [*37*]), where thousands of mobile elements have been studied, 50 % of the insertions were slope specific, with many of them disrupting coding sequences of genes for cognition, olfaction, and thermoregulation, e.g., genes associated with adaptation and speciation. Therefore, each test of a TE is an independent test. Consequently, we concluded that we cannot generalize about the adaptation of TE’s. We can only draw independent conclusions about the adaptation of each individual TE.*

*Our colleagues expected that the same TE’s will be adaptive in both global (=macro) and local (=micro) comparisons. The reviewer ignored our argument that the ecologies differ in both comparisons. In the global comparison both climates are wet, while the warm slope of into “Evolution Canyon” is much DRIER (=SAVANNOID) then the HUMID tropical climates in the global study. Therefore, we expect only partial similarity between the adaptive TE’s of both studies. Moreover, in the article we mentioned one TE, which is significantly adaptive in both continents,* i.e. *Australia and America, but in the opposite directions.*

*Our colleagues regarded “non-significant” as non-adaptive. If the power of the test is not enough there is only one possible conclusion: To increase the sample size, hence to increase the power. Only then has the conclusion “non-significant” a clear meaning. Because of the existing migration we need a larger sample size than in the continental studies. The probability of >95 % is based on the rejection of the hypothesis and not on its acception.*

*In sum, our decision is based on the distribution,* i.e. *on the gaps between the groups of small and large differences, especially the large gap between the TEs higher on the NFS, the observed migration of the flies between the slopes in “Evolution Canyon”, which doesn’t allow drift to be an explanation, and on all the differences between micro- and macro- studies. In addition, we rejected significantly the hypothesis of drift. Because of all that, we think we don’t need to change our title and abstract.*

### 2^nd^ round reviewer comments

#### **Reviewer’s report 1** (2^nd^ round comments)

Eugene Koonin, NCBI, NLM, NIH, United States of America.

**Reviewer comments:** I have no further issues with this manuscript.

**Authors’ response:***We thank Dr Eugene Koonin for agreeing to review and to publish our manuscript.*

#### **Reviewer’s report number 2** (2^nd^ round comments)

Limsoon Wong, NUS, Singapore.

**Reviewer comments**

1. The argument presented by the authors is still quite confused and does not tell a sufficiently sharp and complete story. The null hypothesis concerning the gap is not stated clearly and properly (page 11). Earlier on, the authors say that, under genetic drift, they expect the number of TEs exhibiting low differences should be more than those exhibiting high differences. Therefore to show that the gap is not caused by genetic drift, it is not proper to use 1/3 as the probability of a TE being in the gap region due to genetic drift, as this does not take into account the null hypothesis of genetic drift (the probability decay wrt size of the difference). Yet, the way the authors has presented their argument makes the flat distribution to be their null hypothesis, rather than genetic drift. So this argument is confusing. Moreover, the probability calculation presented in the manuscript was incorrect.
  I am not an evolution biologist. So I don’t know what is reasonable here. But I would rephrase the authors’ argument as follows: Under genetic drift, one expects the probability of TEs exhibiting low/medium/high differences should be x/y/z, with x > y > z and x + y + z = 1. Although we don’t know what the values of x/y/z are, at the most extreme we can assume them to be all very close to 1/3. So the highest number of high-difference TEs that we expect under genetic drift is 1/3 * 28 = ~9, while the observed is 10. So this is pretty close. In fact, the probability of getting 10 or higher number of high-difference TEs is sum{k > = 10} 28Ck *(1/3)^k * (2/3)^(28-k) = ~0.46, which is not significant. I.e., you cannot reject genetic drift if the probabilities x/y/z are close to 1/3 each. You may still be able to reject it under a more extreme decay of x/y/z, but you will need to find these values and justify them.
2. Another concern is the justification given (at top of page 13) for why the multiple corrections of Gonzalez et al. should be done differently. It is not a sound justification from a statistical point of view.
3. Also, I still have concern on how the range of differences is cut into low/medium/high. A strong biological justification is still missing, though the assumptions are stated more clearly now.

**Authors’ response:***We thank Professor Limsoon Wong for agreeing to review and to publish our manuscripts.*

1. *The reviewer tested the partition into 3 groups of TEs and got a non-significant deviation from a flat distribution. We got an entirely different result, by analysing the 3 groups of the absolute values (Fig.*5*). The 3 groups in Fig.*5*(the absolute values) are 14 small, 3 intermediate and 11 large differences between the slopes. That pattern is very significantly different from 1/3 - 1/3 - 1/3 (chi square = ~6.8). But, this test is irrelevant for us, because:*
  *We have a biological explanation for the small and the large. The intermediate are not a third group. We need a larger sample in order to decide to which group they belong. Fortunately, there are no intermediate among the 11 TEs, higher on the ES. In Fig.*6*the large and clear gap is seen in the left side of the distribution, We tested only the creation of that large gap. Our null hypothesis is that the gap is created by chance. The probability for that is P = (2/3)*^*11*^ *= 0.012.*
  *If this gap is not created by chance (= drift), than it separates significantly between those TEs that overrule migration (large differences) and those TEs for which migration overruled the selection, if it exist at all (small and temporary differences).*
  *In the right side of Fig.*6*we didn’t conduct any test, because there was no significant large gap on the AS.*
2. *First, in the multiple comparison correction Gonzales et al.* [1] *ignored the partition of the TEs. Only 18 are supposed to be adaptive. The other 10 are from neutral families, and their inclusion may cause an over correction.*
  *Second, if the tests are repetitions, then a single conclusion is drawn for all repetitions. But, if the tests are independent hypothesises, then if the result is not significant, the sample size needs to be increased in order to achieve significance.*
3. *Biologically we have only 2 groups: small and large differences. The gap in the distribution of the differences may separate them. But note that the answer is valid ONLY for each TE, in the specific climate and environment, regulating the same locus. Therefore, in order to get a final conclusion for each TE, we must continue the study and increase the sample size.*

#### **Reviewer’s report number 3** (2^nd^ round comments)

Fyodor Kondrashov, Center for Genomic Regulation, Spain.

**Reviewer comments:** In this manuscript, as well as in the back-to-back submission (Gonzalez et al.), the authors are analysing the data collected at a very interesting ecological site. The potential strengths of the location and the general concept of the study notwithstanding, the manuscript has substantial weaknesses. Nevertheless, jointly the two papers may, hopefully, inspire debate and the acquisition of better data. At this point, after two previous revisions, I think it is best to have the papers speak for themselves. The only point I think that is relevant for the reader is that the data that it as heart of the debate appears much less voluminous than actual debate. In the age of genomics this could be, and should have been, rectified with relative ease.

**Authors’ response:***We thank Dr. Fyodor Kondrashov for agreeing to review and to publish our manuscript. We think that in the study of our collaborators they didn’t take into account the differences between the****micro****(EC) and the****macro****(continents) studies. Therefore, the experiment in EC has been conducted without enough power and the result of “non-significant” has two alternative meanings: or the significance has not been recognized because there was not enough power or there is really no difference. Only additional research can solve this dilemma. Therefore we concluded that in order to finish the study we need to increase the sample size. Moreover. If the hypothesis is about adaptation than it is about an interaction between the organism and the environment and the answer is valid only in time and location the adaptation has been measured. I don’t see how the knowledge of genomics help to solve the problem and why our debate will be solved.*

## Additional files

Additional file 1: Table S1. Populations and strains analyzed at EC, in this work and the location of the inversions. (DOC 53 kb) Additional file 2: Table S2. TE frequency estimates in stations NFS6 and SFS2 and in each slope. The line separeites between the adaptive families (above line) and the neutral families (below line). The data is also included in **Table 3.** (DOC 60 kb)

## Abbreviations

AS: African slope
EC: Evolution Canyon
ES: European slope
MS: Manuscript
Mt: Mountain
NFS: North facing slope
SFS: South facing slope
TE: Transposable element
