# Supplementary material for: Putative adaptive inter-slope divergence of transposon frequency in fruit flies (Drosophila melanogaster) at “Evolution Canyon”, Mount Carmel, Israel
Source: Biol Direct. 2015 Oct 14;10:58. doi: 10.1186/s13062-015-0074-5 (PMC4604623; doi:10.1186/s13062-015-0074-5)
Supplement: Additional file 1: Table S1. — Populations and strains analyzed at EC, in this work and the location of the inversions. (DOC 53 kb) [file 13062_2015_74_MOESM1_ESM.doc]

**Supplementary Table 1. Populations and strains analyzed at EC,** in this work

| **Population** | **Strains** | **Inversions present** |
| --- | --- | --- |
| North-Facing Slope population 5 (NFS5) | NFS5 27 |  |
|  | NFS5 28 |  |
|  | NFS5 29 | In(2L)t |
|  | NFS5 30 |  |
|  | NFS5 31 | In(2L)t |
|  | NFS5 32 |  |
| North-Facing Slope population 6 (NFS6) | NFS6 33 |  |
|  | NFS6 34 | In(2L)t |
|  | NFS6 35 |  |
|  | NFS6 36 |  |
|  | NFS6 37 |  |
|  | NFS6 38 | In(3R)P |
|  | NFS6 39 |  |
|  | NFS6 40 | In(2L)t |
|  | NFS6 41 | In(2L)t, In(3L)P |
|  | NFS6 42 |  |
|  | NFS6 43 |  |
|  | NFS6 44 | In(2L)t |
|  | NFS6 45 |  |
|  | NFS6 46 |  |
| South-Facing Slope population 1 (SFS1) | SFS1 17 |  |
|  | SFS1 18 | In(2L)t, In(3L)P |
|  | SFS1 19 | In(2L)t |
|  | SFS1 20 | In(2L)t |
|  | SFS1 21 |  |
|  | SFS1 22 | In(2L)t |
|  | SFS1 23 |  |
|  | SFS1 24 | In(3L)P |
|  | SFS1 25 |  |
|  | SFS1 26 |  |
| South-Facing Slope population 2 (SFS2) | SFS2 1 |  |
|  | SFS2 2 |  |
|  | SFS2 3 |  |
|  | SFS2 4 |  |
|  | SFS2 5 |  |
|  | SFS2 6 |  |
|  | SFS2 7 |  |
|  | SFS2 8 |  |
|  | SFS2 9 |  |
|  | SFS2 10 | In(3L)P |
|  | SFS2 11 | In(3R)P, In(3L)P |
|  | SFS2 12 |  |
|  | SFS2 13 | In(2L)t, In(3L)P |
|  | SFS2 14 | In(2L)t |
|  | SFS2 15 | In(2L)t, In(3R)P |
|  | SFS2 16 | 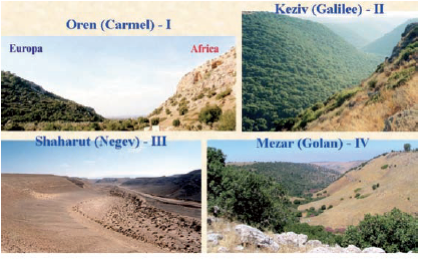In(3L)P |
