# Supplementary material for: Putative adaptive inter-slope divergence of transposon frequency in fruit flies (Drosophila melanogaster) at “Evolution Canyon”, Mount Carmel, Israel
Source: Biol Direct. 2015 Oct 14;10:58. doi: 10.1186/s13062-015-0074-5 (PMC4604623; doi:10.1186/s13062-015-0074-5)
Supplement: Additional file 2: Table S2. — TE frequency estimates in stations NFS6 and SFS2 and in each slope. The line separeites between the adaptive families (above line) and the neutral families (below line). The data is also included in Table 3. (DOC 60 kb) [file 13062_2015_74_MOESM2_ESM.doc]

**Supplementary Table 2.** TE frequency estimates in stations NFS6 and SFS2 and in each slope

|  |  | NFS populations | |  | SFS populations | |  |
| --- | --- | --- | --- | --- | --- | --- | --- |
| **Flybase ID** | **Family** | **NFS 6** | **NFS 5+6** |  | **SFS 2** | **SFS 1+2** |  |
| FBti0018880 | Bari1 | 0.71 | 0.70 |  | 0.69 | 0.69 |  |
| FBti0019056 | pogo | 0.81 | 0.84 |  | 0.75 | 0.79 |  |
| FBti0019065 | pogo | 0.73 | 0.76 |  | 0.81 | 0.73 |  |
| FBti0019144 | Rt1b | 0.25 | 0.21 |  | 0.11 | 0.06 |  |
| FBti0019164 | X-element | 0.45 | 0.39 |  | 0.59 | 0.58 |  |
| FBti0019170 | F-element | 0.39 | 0.38 |  | 0.38 | 0.38 |  |
| FBti0019372 | S-element | 0.25 | 0.25 |  | 0.44 | 0.37 |  |
| FBti0019386 | invader4 | 0.46 | 0.48 |  | 0.25 | 0.32 |  |
| FBti0019430 | Doc | 0.96 | 0.98 |  | 1.00 | 0.98 |  |
| FBti0019443 | Rt1b | 0.36 | 0.35 |  | 0.44 | 0.44 |  |
| FBti0019624 | hopper | 0.79 | 0.75 |  | 0.50 | 0.54 |  |
| FBti0019627 | pogo | 0.61 | 0.66 |  | 0.40 | 0.48 |  |
| FBti0019679 | 1731 | 0.85 | 0.89 |  | 0.89 | 0.87 |  |
| FBti0019747 | F-element | 0.14 | 0.15 |  | 0.19 | 0.21 |  |
| FBti0020042 | jockey | 0.29 | 0.31 |  | 0.36 | 0.32 |  |
| FBti0020046 | Doc | 0.18 | 0.21 |  | 0.43 | 0.43 |  |
| FBti0020091 | Rt1a | 0.92 | 0.87 |  | 1.00 | 0.93 |  |
| FBti0020119 | S-element | 0.27 | 0.34 |  | 0.39 | 0.34 |  |
| FBti0018879 | BS | 0.85 | 0.86 |  | 0.53 | 0.65 |  |
| FBti0019079 | BS | 0.00 | 0.00 |  | 0.13 | 0.08 |  |
| FBti0019133 | BS | 0.72 | 0.69 |  | 0.86 | 0.89 |  |
| FBti0019165 | BS | 0.54 | 0.43 |  | 0.63 | 0.58 |  |
| FBti0019604 | BS | 0.32 | 0.33 |  | 0.34 | 0.34 |  |
| FBti0019771 | 1360 | 0.43 | 0.40 |  | 0.37 | 0.40 |  |
| FBti0020056 | BS | 0.00 | 0.03 |  | 0.07 | 0.07 |  |
| FBti0020057 | BS | 0.68 | 0.65 |  | 0.46 | 0.48 |  |
| FBti0020125 | BS | 0.54 | 0.53 |  | 0.50 | 0.50 |  |
| FBti0020155 | 1360 | 0.64 | 0.63 |  | 0.81 | 0.71 |  |
